# Feasibility and Acceptability of a Text Message–Based Intervention to Reduce Overuse of Alcohol in Emergency Department Patients: Controlled Proof-of-Concept Trial

**DOI:** 10.2196/17557

**Published:** 2020-06-04

**Authors:** Elizabeth Burner, Mark Zhang, Sophie Terp, Kelsey Ford Bench, Joshua Lee, Chun Nok Lam, Jesus R Torres, Michael Menchine, Sanjay Arora

**Affiliations:** 1 Department of Emergency Medicine Keck School of Medicine University of Southern California Los Angeles, CA United States; 2 Los Angeles County + University of Southern California Medical Center Los Angeles, CA United States; 3 Schaeffer Center for Health Policy and Economics University of Southern California Los Angeles, CA United States; 4 School of Medicine University of California San Francisco San Francisco, CA United States

**Keywords:** mhealth, alcohol misuse, emergency department, alcohol intervention

## Abstract

**Background:**

Emergency department (ED) patients have high rates of risky alcohol use, and an ED visit offers an opportunity to intervene. ED-based screening, brief intervention, and referral to treatment (SBIRT) reduces alcohol use and health care costs. Mobile health (mHealth) interventions may expand the impact of SBIRTs but are understudied in low-resource ED populations.

**Objective:**

The objective of this study was to assess the feasibility of and patient satisfaction with a text-based mHealth extension of an ED screening program to reduce risky alcohol use in low-income, urban patients.

**Methods:**

Research assistants screened a convenience sample of ED patients in person for risky alcohol use via the Alcohol Use Disorders Identification Test (AUDIT). Patients who reported AUDIT scores ≥8 and <20 were informed of their AUDIT score and risk. RAs invited patients with SMS text message–capable phones to receive mROAD (mobilizing to Reduce Overuse of Alcohol in the ED), an SMS text message–based extension of the ED screening program. mROAD is a 7-day program of twice-daily SMS text messages based on the National Institutes of Health’s Rethinking Drinking campaign. Participants were allocated to a control group (daily sham text messages without specific guidance on behaviors, such as “Thanks for taking part!”) or to the mROAD intervention group. Patients were interviewed at 30 days to assess acceptability, satisfaction, and changes in drinking behavior. Satisfaction was examined descriptively. Pre and post measurements of drinking behaviors and motivation were compared, as were differences in change scores between the intervention arms.

**Results:**

Of 1028 patients screened, 95 (9.2%) exhibited risky alcohol use based on AUDIT, and 23/95 (24%) of those patients did not own an SMS text messaging–capable phone; this left 72/95 (76%) eligible patients. Among eligible participants, 48/72 (67%) agreed to enroll; 31/48 (65%) achieved follow-up (18/24 (75%) in the intervention group and 13/24 (55%) in the control group). Participants who completed follow-up reported high satisfaction. Changes in behavior were similar between the arms. Overall, the number of drinking days reported in the prior 30 days decreased by 5.0 (95% CI 1.7-8.3; *P*=.004), and the number of heavy drinking days decreased by 4.1 (95% CI 1.0 to 7.15, *P*=.01). Patients reported an 11-point increase (95% CI 2.6-20, *P*=.01, 10% overall increase) in motivation to change alcohol use via the Change Questionnaire. The were no statistical differences in drinking days, heavy drinking days, or motivation to change between the arms.

**Conclusions:**

The mROAD trial was feasible. Over three-quarters of ED patients with risky alcohol use owned a text message–capable phone, and two-thirds of these patients were willing to participate; only 1 patient opted out of the intervention. Although 35% of patients were lost to follow-up at 30 days, those patients who did follow up had favorable impressions of the program; more than 90% reported that SMS text messages were a “good way to teach,” and 89% of intervention arm participants enjoyed the program and found that the messages were motivating. Both the mROAD and sham message groups showed promising changes in alcohol use and motivation to change. mROAD is a feasible intervention that may reduce rates of risky alcohol use in ED patients.

**Trial Registration:**

ClinicalTrials.gov NCT02158949; https://clinicaltrials.gov/ct2/show/NCT02158949

## Introduction

Alcohol-related harm is responsible for an estimated $249 billion in yearly economic costs in the United States [1,2]. Efforts to reduce risky alcohol use are consistently cost-effective [3,4]. Unfortunately, only 10%-15% of patients who require treatment for substance use actually receive it [5,6]. Screening, brief intervention, and referral to treatment (SBIRT) is a cost-effective strategy to intervene in risky alcohol use. SBIRTs consist of screening (screening patients’ alcohol use with standardized tools), brief intervention for moderate risk screens (informing patients of the health risks posed by their current use and motivational interviewing techniques to encourage behavior changes), and referral to treatment for high-risk screens [7-9]. SBIRTs decrease ED utilization, health care costs, and risky alcohol use in multiple settings [10-14].

While SBIRTs can reduce risky alcohol use and decrease health care costs, many EDs still do not deploy them. Barriers of competing time priorities, lack of provider training in addiction medicine, and social stigma limit broad implementation of SBIRTs [15-18]. A brief intervention of 5-20 minutes per at-risk patient is recommended, which may exceed the total time a busy ED provider has to spend with a patient on that visit [19]. Strategies to reduce the provider time needed to deliver SBIRTs may increase their use and implementation.

Mobile health (mHealth) is a strategy that can be implemented to decrease the provider time required to deliver SBIRTs. mHealth includes the use of phone applications, SMS text messaging, and web-linked portals to provide public health interventions and clinical care. mHealth has been successfully used in the past to improve outcomes in a wide range of health issues, including substance use [20-22]. mHealth-based interventions for ED patients with risky alcohol use have been successful with young adult binge drinkers as well as with injured patients in New Zealand [23,24]. However, there is limited data on the role of mHealth in SBIRTs in other populations, including low-income, urban EDs and non–English-speaking patients. Previous work with low-income, urban ED patients has shown these patients to be ready to accept mHealth interventions and to own mobile phones capable of receiving these interventions [25-29]. However, it is not known if patients with high-risk alcohol use have the same access to mobile technology.

To understand the feasibility of an mHealth extension of an SBIRT in a low-income, predominantly non–English-speaking population, we conducted a proof-of-concept trial in the ED of an urban, academic safety net hospital. Patients received screening and notification of risk in the ED and were allocated to either an intervention group, which received twice daily theory-driven SMS text messages, or an active control group, which received daily nonspecific text messages for seven days. We collected feasibility data, perceptions of acceptability, and preliminary efficacy data 30 days after the intervention ended.

## Methods

### Study Design and Location

This quasiexperimental trial took place at an urban Level 1 trauma medical center (Los Angeles County + University of Southern California Medical Center) in Los Angeles, CA. Local institutional review board approval was obtained prior to the beginning of the study. The study was registered with ClinicalTrials.gov (NCT02158949).

### Screening and Recruitment

Trained research assistants reviewed the ED electronic tracking board in real time between 7 AM and 11 PM over the course of 3 months (May through July 2014). Adult ED patients were approached in person for initial screening unless precluded by a clinical condition, language barrier (any language other than English or Spanish), or other inability to verbally consent to screening. Patients were screened using a tablet-based survey unless they preferred to have the survey read aloud to them by the research assistant. Patients were screened on level of alcohol use via the Alcohol Use Disorders Identification Test (AUDIT) developed by the World Health Organization (WHO) [30] as well as on mobile phone ownership and mobile technology use via questions developed by the Pew Center [25]. Patients with SMS text messaging–capable phones who were at risk for alcohol use disorders (AUDIT scores ≥ 8 but <20) were recruited to the study regardless of the reason for their visit [31]. AUDIT scores of 8-19 reflected patients who might benefit most from SBIRTs, while those with scores 20 and above required more intense intervention [32]. The AUDIT has excellent retest reliability, with a mean Cronbach α=.8 in a review of 10 studies [33]. Standard AUDIT scoring per WHO organization instructions was used [30].

At recruitment, RAs informed the patient that they were at risk for hazardous alcohol use and invited them to receive SMS text messages upon enrolling. Patients were offered an incentive of a $10 gift card if they choose to enroll to offset the cost of receiving the SMS text messages. Patients were given both a verbal explanation of the project and a copy of the informed consent form.

After agreeing to participate, participants were sequentially assigned to either the active control group or the intervention group. Next, patients were registered in the mHealth platform, which sent automated, unidirectional, broadcast SMS text messages for 1 week. Patients selected their preferred language for text messages as English or Spanish. Patients were not required to text a response to be enrolled.

### Measures

On enrollment, patients reported their alcohol use by responding to two questions. The first question was a general alcohol use question: “Please think back over the last month. How many days did you drink?” The second question was a gender-based assessment of heavy drinking: “Over the last month, how many days did you drink heavily?” (“Heavy drinking” was defined for women as more than 3 drinks in one day and for men as more than 4 drinks in one day.) Patients were defined as everyday drinkers or binge drinkers based on their responses to the initial AUDIT screen. Additionally, participants reported their desire to change via the Change Questionnaire applied to risky alcohol use [34-36]. The Change Questionnaire consists of 12 statements on a patient’s belief of the importance of change, commitment to change, and ability to change; its Cronbach α=.86 when applied to alcohol use [37]. AUDIT scores and mobile phone ownership data were taken from the screening data.

### Intervention

The mROAD intervention was entirely SMS text message–based, given the low access to advanced mobile technology in this population [27]. mROAD is a unidirectional, automated system; it was set to start the day after a patient enrolled in the trial. Messages were delivered on a Health Insurance Portability and Accountability Act (HIPAA)-compliant system that was compatible with the local pay-as-you-go cellular plans that are popular in this patient population. The mROAD intervention was developed in English and then translated to Spanish, with back translation by two native Spanish speakers to ensure a clear translation. Patients received either the mROAD intervention (described below) or a week of sham SMS text messages so that patient willingness to receive messages could be assessed in both arms.

**Figure 1:**
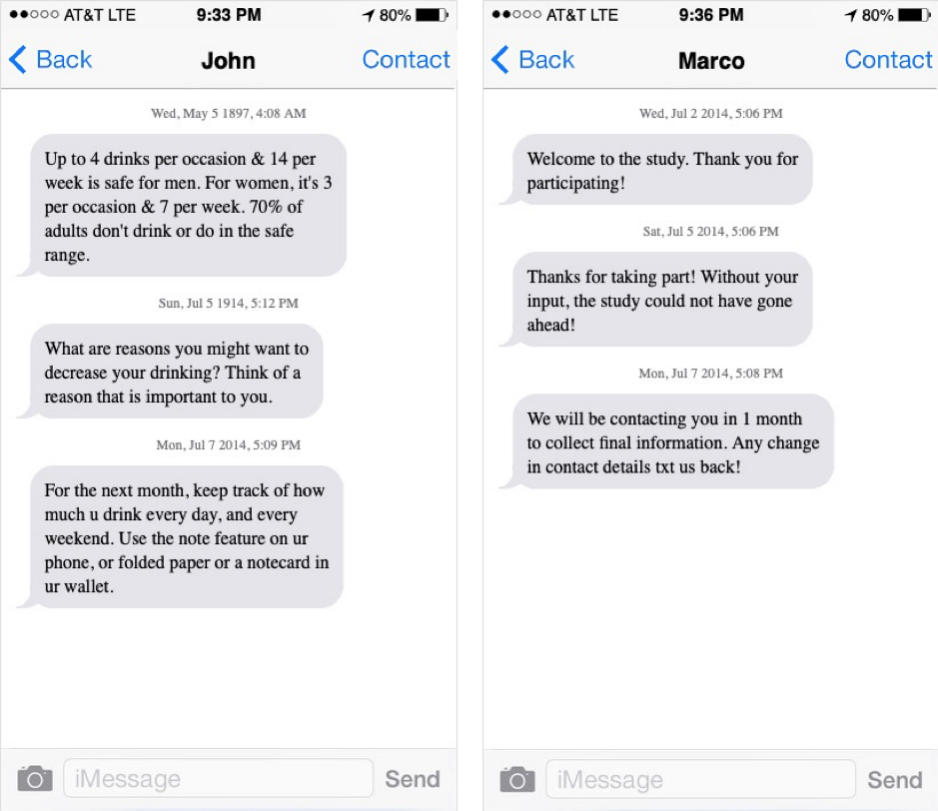
Example mROAD and sham messages. The images were generated at fakeiphonetext.com.

The active control arm (sham) received a sham SMS text message greeting daily (eg, “Thanks for taking part!”), while the intervention group (mROAD) received two text messages about alcohol use daily for 7 days. The intervention SMS text messages were adapted from the National Institutes of Health publication *Rethinking Drinking* [38]*.* The messages were shortened to fit the character limit of SMS text messages. The selected content included the consequences of drinking, motivational statements, and resources on how to obtain help to reduce drinking. Social norms theory and motivational interviewing strategies were emphasized, as supported by systematic reviews of the literature [39]. For example, social norms theory–based messages described normal drinking behavior, while motivational interviewing–based messages prompted participants to set a goal and write it down or type it out (see Figure 1 for example mROAD and sham messages).

### Follow-up Procedures

Patients were contacted 30 days after the intervention to complete a telephone follow-up assessment. Research assistants were blinded to the treatment group at time of follow-up. Patients reported their days of drinking alcohol over the past month, days of heavy drinking over the past month, and desire to change their alcohol-drinking behavior by responding to the same questions asked at enrollment but without visual prompts. Patients in each group also completed a brief acceptability questionnaire. At the end of the trial, we collected the service records of the mobile health platform to ensure that the messages were delivered to each participant as scheduled.

### Outcomes and Analysis

Feasibility was defined as >60% of eligible patients consenting and enrolling in the program and achieving 60% follow-up with participants. Previous studies with SBIRTs among ED patients yielded enrollment rates of 38%-87%, with follow-up rates between 49% and 89% [14,24,40]. For the few SMS text message–based alcohol interventions from the ED, follow-up rates have been between 75% and 82% [23,41,42]. However, previous work at our study site showed a maximum telephone follow-up rate of 70% for all comers [43]. To account for the lower anticipated follow-up rates for a low-income, non–English-speaking ED population in addition to patients with risky alcohol use, we determined 60% to be an acceptable follow-up rate.

We defined acceptability as greater than 90% of patients completing the 7 days of text messages without opting out by review of the mHealth platform service records. Our secondary acceptability outcome benchmark was 75% of participants agreeing with each statement in a brief, locally developed acceptability questionnaire. We also reported preliminary efficacy results with changes in days drinking alcohol, days heavily drinking, and desire to change drinking behavior. Preliminary efficacy results were compared to baseline and between groups with two-sample *t* tests without assumption of equal variance.

## Results

### Feasibility Outcomes

**Figure 2:**
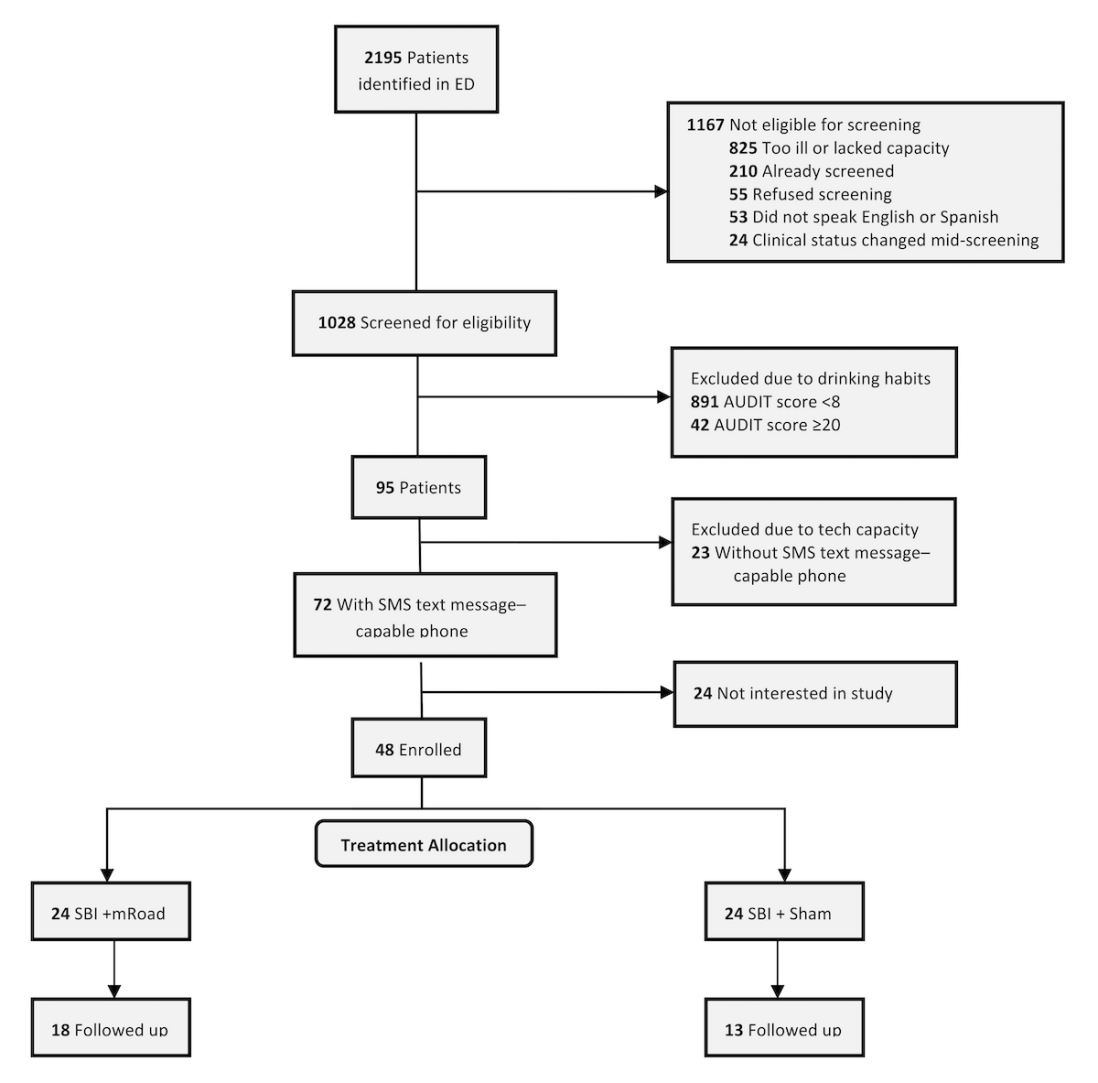
Flow diagram of the study.

2195 patients were identified by real time electronic tracking board review; 1167 could not be screened (see Figure 2 for exclusion reasons; the most common was that the patient was too ill to consent, n=825, 70.6%). Of the 1028 ED patients screened for alcohol use, 95 (9.2%) exhibited risky alcohol use based on AUDIT, and 72 (76%) of those patients owned an SMS text messaging–capable phone. Two-thirds of eligible patients (48/72, 67%) consented and were enrolled and registered in the mobile health platform.

The patients enrolled in the study were predominately male, Latino, and aged 30-39 years (see Table 1 for the complete study population characteristics). More than half spoke Spanish primarily. Compared to patients in the mROAD arm, patients allocated to the sham message intervention group had lower self-reported rates of mental illness (13% vs 25%) and higher numbers of days drinking alcohol and days drinking heavily in the prior month. These baseline differences were not statistically significant.

Nearly two-thirds (31/48, 65%) of enrolled patients were successfully reached for follow-up; follow-up was higher in the intervention group (18/24, 75%) than in the control group (13/24, 54%). More patients in the intervention group reported receiving messages (17/18, 94%) than patients in the active control group (11/13, 85%).

**Table 1:** Baseline characteristics of the study participants (N=48).

Characteristic	Sham group (n=24)	mROAD^a^ group (n=24)
Age (years), mean (SD)	35.4 (10.6)	38.8 (13.5)
Male gender, n (%)	21 (88)	19 (79)
Spanish speaking, n (%)	14 (58)	14 (58)
Latino ethnicity, n (%)	21 (88)	17 (71)
Self-report of mental illness, n (%)	3 (13)	6 (25)
Number of days drinking alcohol last month, mean (SD)	11.5 (8.4)	7.6 (8.6)
Number of days drinking heavily^b^ last month, mean (SD)	5.5 (7.13)	4.9 (7.2)
Motivation to change drinking (score 0-120), mean (SD)^c^	89.3 (33.8)	88.8 (6.7)

^a^mROAD: mobilizing to Reduce Overuse of Alcohol in the emergency Department.

^b^Drinking heavily was defined as >3 standard-sized drinks per episode for women and >4 standard-sized drinks per episode for men.

^b^Measured by the Change Questionnaire.

### Acceptability Outcomes

Overall, acceptance of the intervention was high among patients who were followed up, and all acceptability benchmarks were achieved. Review of the mHealth platform records indicated that only 1 patient in the intervention arm opted out of the messages; the patient opted out after the first intervention message was sent. All other patients received the full 7 days of messages. Of the 31 patients assessed at follow-up, more than 90% agreed that using SMS text messages was a “good way to teach,” nearly four-fifths reported that the number of messages per day was “just right,” and more than half wanted the messages to continue (see Table 2).

There were differences in acceptability between the two arms. Patients in the intervention arm had higher acceptance of the program than patients in the sham arm. More patients in the intervention arm enjoyed the program, were willing to recommend it to friends and family, were motivated by the messages, and perceived that the messages came at the right time of day.

**Table 2:** Acceptability as indicated by participants’ agreement with the following statements at 30 day follow-up, n (%).

Statement	Sham group (n=13)	mROAD^a^ group (n=18)
Using texts is a good way to teach	12 (92)	17 (94)
I enjoyed the mROAD program	9 (69)	16 (89)
I would like the messages to continue	7 (54)	10 (56)
I would recommend mROAD to family and friends	9 (69)	16 (89)
I was motivated by the mROAD messages	9 (69)	16 (89)
The messages came at a good time for me	9 (69)	15 (83)
The number of messages per day was just right	10 (77)	14 (78)

^a^mROAD: mobilizing to Reduce Overuse of Alcohol in the emergency Department.

### Preliminary Efficacy Outcomes

Patients in both arms reported increased motivation to change drinking behavior, decreased days drinking any alcohol, and decreased days drinking heavily (see Table 3). Overall, participants reported increased motivation to change alcohol use, with an 11-point increase (95% CI 2.6-20, *P*=.01, 10% overall increase) on the Change Questionnaire. The number of reported drinking days in the prior 30 days decreased by 5 (95% CI 1.7-8.3, *P*=.004 and heavy drinking days decreased by 4.1 (95% CI 1.0-7.15, *P*=.01). The differences in the changes between the arms were not significant; however, the sham message arm overall trended toward larger improvements.

**Table 3:** Mean changes in drinking habits and desire to change for each group and combined among participants who were followed up.

Variable	Sham group (n=13)		mROAD^a^ group (n=18)		Combined (n=31)			Differences between groups^b^	
	Mean (95% CI)	*P* value	Mean (95% CI)	*P* value	Mean (95% CI)	*P* value	Mean (95% CI)		*P* value
Decrease in days drinking alcohol	8.5 (3.0 to 13.9)	.005	2.5 (–1.7 to 6.7)	.23	5 (1.7 to 8.3)	.004	–5.9 (–0.6 to 12.5)		.07
Decrease in days drinking heavily^c^	5.6 (–0.1 to 11.4)	.05	3 (–0.7 to 6.7)	.11	4.1 (1.0 to 7.15)	.01	–0.6 (3.6 to 4.8)		.78
Increased motivation to change (score 0-120)^d^	16.6 (­–1.6 to 34)	.07	7.6 (–1.7 to 17)	.01	11.2 (2.6 to 20)	.01	8.9 (8.7 to –26.6)		.31

^a^mROAD: mobilizing to Reduce Overuse of Alcohol in the emergency Department.

^b^*t* tests were used to compare pre-post measures and between-group differences.

^c^Heavy drinking was defined as >3 standard-sized drinks per episode for women and >4 standard-sized drinks per episode for men.

^d^Measured by the Change Questionnaire.

## Discussion

We conducted this proof-of-concept trial to assess the feasibility of an mHealth extension of an ED-based SBIRT to decrease risky alcohol intake among low-income, urban, predominantly Latino ED patients. We found that screening and enrollment was feasible; more than 60% of patients with risky alcohol intake owned an SMS text message–capable phone, and more than 60% of eligible patients agreed to participate. In addition, patients accepted the mROAD intervention; only 1 patient left the intervention, and more than 80% of intervention group patients who were followed up reported favorable perceptions of mROAD. Preliminary efficacy results from the combined groups indicate that mHealth extensions of SBIRTs should be tested in larger and longer trials.

As a proof-of-concept trial, this study shows that mHealth extensions of SBIRTs are feasible in a low-income, urban, predominantly Latino population. As with most alcohol screening programs, the most common reason for ineligibility was not reporting risky levels of alcohol intake. Approximately 30% of patients with risky alcohol use were ineligible due to lack of mobile phone ownership; this rate is higher than in similar interventions with younger, nonminority populations [44]. As the mobile capacity of low-income Latino patients continues to increase, the gap between patient capacity and intervention delivery will narrow [26,45]. Additionally, more than two-thirds of eligible patients chose to participate, which is at the high end of mHealth interventions for alcohol use [24,40,44]. Lastly, we maintained an adequate follow-up rate, with two-thirds of patients following up by phone; this indicates that studying this type of intervention is feasible.

In addition to being feasible, this mHealth extension of in-ED screening was acceptable and satisfactory to patients. Patients who received theory-based mROAD messages were more engaged with the study; 75% were reached for follow-up, compared with 54% of patients who received daily sham messages. Patients in both groups found the mHealth extension of in-ED screening to be helpful and motivating. Patients who received theory-based mROAD messages reported generally higher satisfaction with and motivation from the program, and they were more likely to recommend it to a friend or family member. These acceptability results are promising for larger-scale trials and widespread implementation.

While mHealth extensions of in-ED SBIRTs are feasible, most EDs still do not conduct standardized screening and intervention, which limits their implementation [18]. However, promising work using patient self-administered and computer-based screening and notification of risk provides an opportunity to increase the number of patients screened and referred to an mHealth extension of an SBIRT [46-49]. As more EDs move to self-administered screening of behavioral risk factors and social determinants of health via computer, tablet, and mobile device interfaces, it may be possible to formally screen more patients for risky alcohol use [50,51]. Integrating formalized screening for alcohol behaviors increases implementation of screening and SBIRTs [52,53]. Increased screening could provide a larger target population, which could require increased resources at individual institutions. By using mHealth SBIRT strategies in combination with in-ED computer-based, tablet-based, and mobile device–based screening, the scope of SBIRTs can be increased. For clinics and EDs that already screen for risky alcohol use, similar mHealth extensions of screenings and brief interventions would require marginal extra workforce time. mHealth interventions hold potential to create large-scale programs to reduce risky drinking among ED patients without increasing demands on an already overstretched ED workforce.

In our study, patients in the sham message arm reported decreased drinking days, heavy drinking days and increased motivation to change drinking. The patients in the sham message arm started with higher reported drinking days, which correlates with larger decreases in risky alcohol behavior in prior ED-based SBIRTs [54,55]. Additionally, the follow-up rate was lower in the sham message arm; sham message patients who were followed up may be more motivated than the average ED patient. While the difference was not significantly different from the theory-based message arm, there are several possible explanations if this finding is verified in fully powered studies. Patients receiving theory-based messages may become more aware of their drinking habits if they are reminded with messages pertaining to their drinking rather than sham messages alone. As a consequence, they may more accurately report their drinking frequency than the patients in the control group. This study did not have a usual care control group, as all patients were first informed in the ED during their initial contact that they were at risk for alcohol abuse. The minor intervention of daily SMS text messages linked to the ED-based screening may have promoted a change in the patients’ habits.

While this proof-of-concept study is promising, it has several limitations. A strength of this study and of the mROAD program is the demonstration of the potential of a simple, easily scalable, automated system to encourage positive behavior changes; however, the small sample size, quasiexperimental design, and short follow-up period prevent conclusions about sustained behavior changes or differences between patients who received theory-driven vs sham messages. Patients in the sham message and theory-driven mROAD arms had similar reported changes in alcohol use and motivation to change; this indicates that either the sham messages after the in-ED screening and risk notification had beneficial effects alone or that the natural history of an ED visit may include a decrease in alcohol use. Further study of this type of intervention may require a control group with less activation. This study was conducted at a single site, which may limit the generalizability of the feasibility findings. Additionally, the logistics of patient follow-up from an ED-based study that serves a low-income, non–English-speaking population creates potential for biased results due to differential follow-up.

This proof-of-concept study shows that low-income, urban ED patients can feasibly be enrolled in mHealth extensions of ED-based screening and brief intervention programs. We found that patients were willing to participate in mROAD and were accessible for follow-up. mHealth extensions of face-to-face clinical care can extend the impact of an ED visit well beyond the physical confines of the hospital.

## Appendix

Multimedia Appendix 1 CONSORT-eHEALTH checklist (V 1.6.1).

## Abbreviations

AUDIT: Alcohol Use Disorders Identification Test
ED: emergency department
HIPAA: Health Insurance Portability and Accountability Act
mHealth: mobile health
mROAD: mobilizing to Reduce Overuse of Alcohol in the emergency Department
SBIRT: Screening, Brief Intervention, and Referral to Treatment
WHO: World Health Organization
